# Transverse myelitis after SARS‐CoV‐2 infection: Report of two cases with COVID‐19

**DOI:** 10.1002/ccr3.5196

**Published:** 2021-12-18

**Authors:** Soroor Advani, Seyyed Mohammad‐Mahdi Hosseini, Alireza Zali, Davood Ommi, Alireza Fatemi, Reza Jalili Khoshnoud, Farzad Ashrafi

**Affiliations:** ^1^ Neurology Department Shohada Tajrish Hospital Shahid Beheshti University of Medical Sciences Tehran Iran; ^2^ Student Research Committee Department and Faculty of Medicine Shahid Beheshti University of Medical Sciences Tehran Iran; ^3^ Functional Neurosurgery Research Center Shohada Tajrish Neurosurgical Center of Excellence Shahid Beheshti University of Medical Sciences Tehran Iran

**Keywords:** angiotensin‐converting enzyme 2 (ACE2), magnetic resonance imaging (MRI), neurology, plasma exchange, respiratory distress

## Abstract

Transverse myelitis has been reported as a complication of COVID‐19 in recent studies. Here, we report two cases of transverse myelitis related to COVID‐19. Both patients underwent plasma exchange after being treated with antibiotics and corticosteroids which lead to the recovery of one of them.

## Graphical abstract

As COVID‐19 pandemic continues, its neurological manifestations have emerged. Therefore, it is essential for clinicians facing patients with transverse myelitis even without history of fever and cough, to consider COVID‐19 besides other well‐known etiologies.

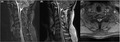

## 1 INTRODUCTION

The coronavirus disease 2019 (COVID‐19) epidemic began in Wuhan, China was caused by the novel severe acute respiratory syndrome coronavirus 2 (SARS‐CoV‐2) and has spread to almost all countries worldwide.^1^ So far, 228,394,572 confirmed cases of coronavirus disease 2019(COVID‐19) had been recorded, leading to 4,690,186 deaths.^2^

COVID‐19 affects multiple organs, leading to multiple manifestations. The most common symptoms of COVID‐19 are fever and cough. COVID‐19 can also cause fatal respiratory distress.^3^ However, given that the COVID‐19 pandemic is evolving, new clinical presentations have been reported among patients.^4^

SARS‐CoV‐2 may enter the cell through angiotensin‐converting enzyme 2 (ACE2) receptors^5^found on various organs, including alveolar epithelial cells, intestinal enterocytes, and arterial and venous endothelial cells.^6^Additionally, SARS‐CoV‐2 may lead to systemic inflammation, which can cause multiple organ dysfunction syndrome (MODS). Therefore, COVID‐19 can cause severe renal impairment, liver damage, and pancreatitis, among other complications. The hypoxia and atheroma rupture caused by COVID‐19 can also cause myocardial infarction and cerebrovascular stroke.^7^ According to autopsy studies of patients with COVID‐19, diffuse alveolar damage, thromboembolism, disseminated intravascular coagulation (DIC), and cerebral vasculitis were common.^8^

SARS‐CoV‐2 invades the CNS through the olfactory bulb, ACE2 receptors on the blood‐brain barrier (BBB), and infected leukocytes cross the BBB. There are also other mechanisms by which SARS‐CoV‐2 can damage the CNS, such as inflammation, ischemia, and hypoxia. COVID‐19 has been shown to cause encephalopathy, encephalitis, meningitis, stroke, Guillain‐Barre syndrome (GBS), and acute polyneuropathy. Recent studies have shown COVID‐19 to be associated with hypogeusia, hyposmia, acute polyneuropathy, and headaches.^9^

Since early in the pandemic, several cases of acute transverse myelitis have been reported in association with COVID‐19 worldwide.^10^, ^11^, ^12^, ^13^, ^14^ In this article, we describe two patients a 47‐year‐old man and a 67‐year‐old woman with transverse myelitis after infection with the SARS‐CoV‐2 virus, hospitalized in Shohada Tajrish Hospital, affiliated with Shahid Beheshti University of medical sciences. We also discussed the presentation of patients, diagnostic and therapeutic approaches we have used, and patients' outcomes and compared our results with those from previous studies.

## 2 PATIENT 1

The 47‐year‐old man was admitted to the emergency room with paraplegia. Symptoms began 12 h before the weakness of the lower limbs with urinary retention. Abrupt paraplegia followed by a dull abdominal and flank pain subsided within hours. The patient also complained of a decreased sensation of lower limbs and trunk. He denied any symptoms in his upper limbs. The patient had fever, cough, and diarrhea 10 days before developing paraplegia. He was diagnosed with COVID‐19 and quarantined at home. His past medical history revealed no findings.

The patient was afebrile with an oxygen saturation of 98% and a respiratory rate of 15 per minute based on a physical examination. Initial neurological examination revealed intact cranial nerves and weakness of lower limbs with medical research council(MRC) score of 0/5 in both proximal and distal. Lower limbs were flaccid, and deep tendon reflexes were absent. There was a decreased sensation of all modalities in lower limbs with a sensory level at T10. Plantar reflexes were neutral, and abdominal reflex was also absent. Upper limb motor, sensory, and cerebellar examinations were normal.

**FIGURE 1:**
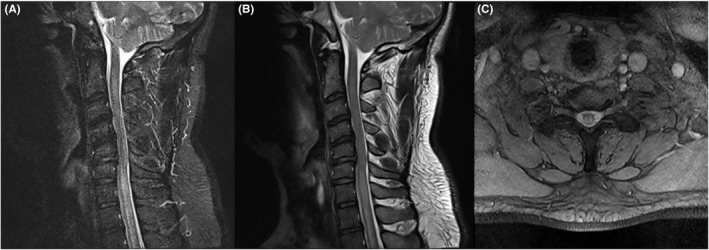
Cervical magnetic resonance imaging of first patient: longitudinally extensive transverse myelitis involving second cervical segment to second thoracic segment of spinal cord. (A) Sagittal section, Short‐TI Inversion Recovery sequence. (B) Sagittal section, T2 sequence. (C) Axial section, T2 sequence: hyperintensity involving more than 2/3 of axial section

Nasopharyngeal swab test for SARS‐CoV‐2 PCR was positive. SARS‐CoV‐2 serology was as follows: IgM: 11.72 IU/ml (>1.1 positive), IgG:18.31 IU/ml (>1.1 positive). Whole spinal magnetic resonance imaging(MRI) showed a longitudinal extensive myelitis(LETM) involving the second cervical to the second thoracic segment of the spine (Figure 1). The lesion had gadolinium enhancement. Lumbar puncture was performed with glucose: 60 mg/dl, protein: 110 mg/dl, and white blood cell: 650(80% polymorphonuclear cells and 20% mononuclear cells). CSF gram stain and culture and serology tests of CSF for herpes zoster, varicella‐zoster, and Epstein‐Barr virus were negative. The patient's serum Aquaporin‐4 antibody was also negative. Autoimmune immunological screening including Lupus anticoagulant, Protein S and C levels, Anti‐Neutrophil Cytoplasmic antibodies, Rheumatoid factor (RF), Anti Cardiolipin, and Anti Beta 2 Glycoprotein were all negative. The patient was diagnosed with COVID‐19‐associated transverse myelitis, but infectious etiology was still considered due to the patient's CSF analysis.

Treatment was started with parenteral antibiotics (ceftriaxone and vancomycin), acyclovir, and five sessions of plasma exchange. Unfortunately, the patient did not respond to the treatment, so he was discharged to a rehabilitation center.

## 3 PATIENT 2

The 67‐year‐old woman was referred from the neurology clinic after experiencing paraparesis for 1 month. Initial symptoms were a sensory level and paresthesia at chest and urinary frequency that progressed to paraparesis, resulting in frequent falls. At the time of the visit, she declared that her symptoms had a worsening nature. She had no symptoms in her upper limbs. She denied having fever, cough, dyspnea, or gastrointestinal symptoms weeks before her symptoms, but her appetite had decreased 3 weeks ago. Her close relatives did not exhibit these symptoms or have confirmed COVID‐19, at least to her knowledge. Her past medical history was unremarkable.

On physical examination, she was afebrile, had no respiratory distress, and her oxygen saturation was 99%. Her general examination was unremarkable. The initial neurologic examination revealed symmetric proximal and distal lower limb weakness with a 4‐/5 MRC score. Deep tendon reflexes were increased symmetrically in the lower limbs. She had no sensory level, and plantar reflexes were downward. Abdominal reflexes were not reliable due to previous pregnancies. Cranial nerves, sensory, and cerebellar examination were unremarkable.

**FIGURE 2:**
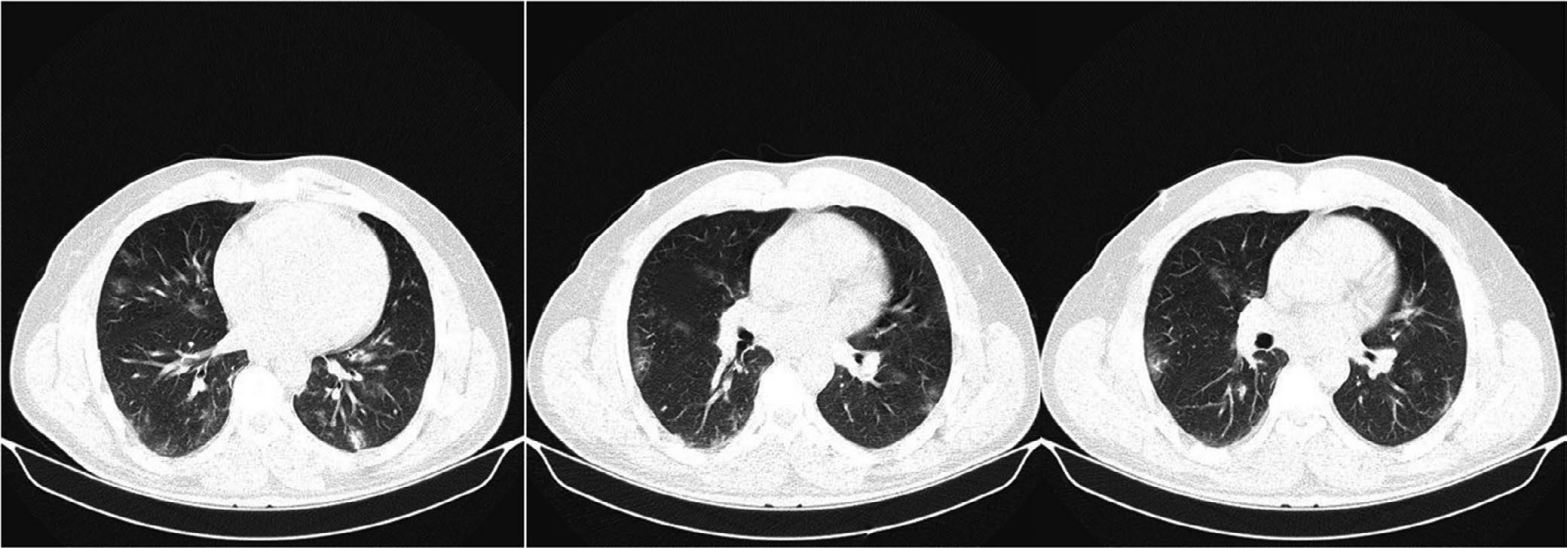
Chest computed tomography with diffuse ground glass hyperdensities involving both lungs. Some non‐significant mediastinal lymph nodes are also detected. These findings are suspicious for COVID‐19

**FIGURE 3:**
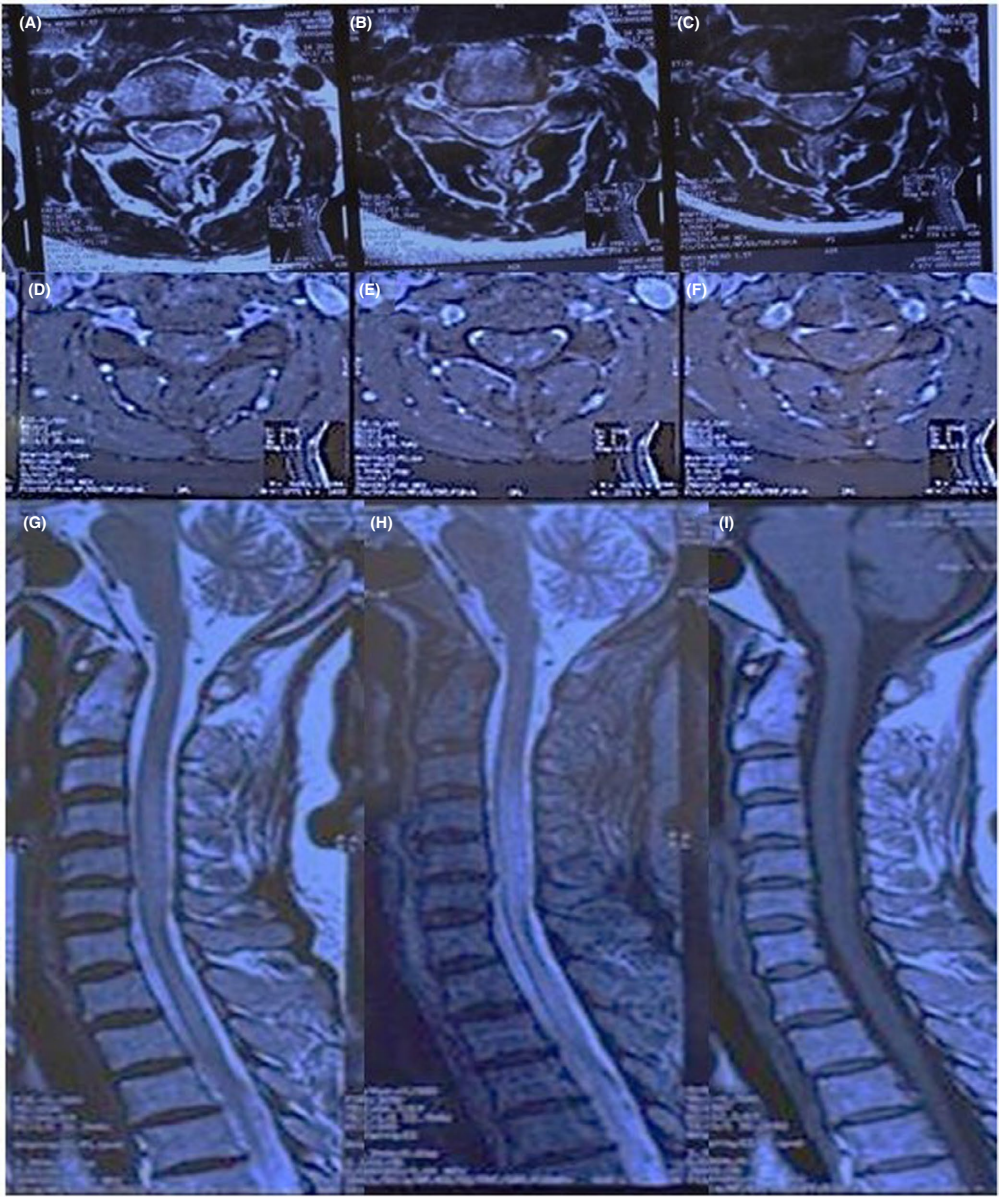
Cervical magnetic resonance imaging of second patient: longitudinally extensive transverse myelitis involving third to sixth cervical segment of spinal cord. (A) Sagittal section, T2 sequence shows hyperintense lesion from C3 to C6. (B) Axial section, T2 sequence: hyperintensity involving more than 2/3 of axial section. (C) Axial section, T1 with gadolinium sequence: faint enhancement of aforementioned lesion

Nasopharyngeal swab test for SARS‐CoV‐2 PCR was negative. SARS‐CoV‐2 serology revealed a positive IgG test (3.58 IU/ml with >1.1 considered as positive) and negative IgM (0.7 IU/ml with >1.1 considered as positive). This result was interpreted as previous exposure to SARS‐CoV‐2. Her chest computed tomography was also suggestive of COVID‐19 (Figure 2). A whole spinal MRI was performed that revealed an LETM involving the third cervical to the sixth cervical segment of the spine. The lesion had gadolinium enhancement (Figure 3). Lumbar puncture was performed with glucose: 80 mg/dl, protein:40 mg/dl, and no cells. Gram stain, culture, varicella‐zoster, and EBV serology of CSF returned negative. The patient's serum Aquaporin‐4 antibody was also negative. Autoimmune immunological screening including Lupus anticoagulant, Protein S and C levels, Anti‐Neutrophil Cytoplasmic antibodies, Rheumatoid factor, Anti‐Cardiolipin, and Anti‐Beta 2 Glycoprotein were all negative. CSF was tested for oligoclonal bands(OCB), which was positive (many bands), and the IgG index was 0.9(positive>0.7).

The patient was diagnosed with transverse myelitis, and after ruling out infectious causes, she was treated with a 5‐day course of parenteral methylprednisolone (1 gram for each session) that did not result in any recovery. She was started on plasma exchange. After five sessions of plasma exchanged, the patient experienced significant recovery. She was fully ambulatory with the lower limbs muscle power of 4+/5 at the time of discharge.

Motor evoked potentials have a limited role in evaluating patients with a clinical picture compatible with myelopathy and a confirmatory imaging. We used neurological examinations to follow‐up with our patients.^15^

## 4 DISCUSSION

This report describes two patients with transverse myelitis following COVID‐19, both of whom had different onset of disease, severity, and response to immunomodulation therapy. The neurological symptoms of patient 1, including severe motor and sensory loss in lower limbs, developed abruptly 7 days after acute symptoms of COVID‐19. However, symptoms of patient 2 evolved rather insidiously, were milder, and unlike patient 1, responded well to treatment.

The symptoms of our cases were similar to those reported in previous studies such as by Kang Zhao et al., Abdelhady et al., and Alketbi et al.^16^, ^17^, ^18^, ^19^ A case reported by Shahali et al. was similar to that of patient one, in which sensory and lower limb motor loss were associated with a flu‐like syndrome without any traumatic injury. Unlike patient 1, Shahali H et al. case's symptoms improved dramatically after IVIg therapy.^20^ There were also similarities between patient two and Sarma et al., and Zachariadis et al. reported cases.^13^, ^21^ Mild symptoms and a favorable response to immunomodulatory treatment in patient two suggest an inflammatory condition, but the absence of CSF reactions such as pleocytosis and increased protein levels refuted this theory.

Aside from the differences between our cases, the location of the lesions was similar in both. Several studies have shown that the most common site of the spinal cord is the cervicothoracic area, which was also seen in both of our patients (cervicothoracic in the first and cervical in the second case).^22^

Transverse myelitis, caused by inflammation of the spinal cord, may be triggered by an infection, post‐infectious immune‐mediated reactions, multiple sclerosis (MS), neuromyelitis optica, systemic autoimmune diseases, or infarction.^23^ Two hypotheses may explain transverse myelitis caused by COVID‐19. A hypothesis suggests that myelitis is a result of the direct viral invasion. It is proposed that SARS‐CoV‐2 invades the brain via ACE2 receptors on brain and spinal glial cells and from vascular endothelium. According to another hypothesis, transverse myelitis develops through the immune response to SARS‐CoV‐2 following infection.^24^ The rapid and acute onset of transverse myelitis in patient 1 supports the first hypothesis. The latter hypothesis can explain the nature of symptoms in patient 2. The patient 2 had been exposed to SARS‐CoV‐2 in the past as her IgG test for COVID‐19 was positive but declared no other noteworthy symptoms attributed to COVID‐19. Therefore, our study suggests that transverse myelitis can occur in patients with COVID‐19 regardless of their respiratory symptoms. In other words, clinicians should consider past exposure to and current infection with SARS‐CoV‐2 as a possible etiology when evaluating patients with transverse myelitis.

Based on how the patients in this report responded to the treatment, there may be a relationship between respiratory symptoms and the prognosis of transverse myelitis. Answering this question will require further research.

This report contains limitations related to the CSF analysis. The PCR was not performed on the CSF of both patients. However, previous studies have revealed that this limitation can likely be overlooked since the PCR would probably be negative.^25^, ^26^ The other limitation is the CSF analysis which raises suspicions about other infectious etiologies. The CSF analysis of patient two did not reveal any abnormalities, except for a positive IgG index and OCB presence. The presence of these markers in patient 2's CSF may indicate inflammatory disorders such as multiple sclerosis (MS) and neuromyelitis optica (NMO). Negative serum Aquaporin‐4 antibody ruled out the diagnosis of NMO, and no imaging findings attributed to MS were found. Nevertheless, we cannot exclude NMO and MS, and following up with patient two would be more confident in a diagnosis.

## 5 CONCLUSION

Transverse myelitis has been reported as a complication of COVID‐19 in several case reports. Despite the limited data, it could be suggested that SARS‐CoV‐2 invades the CNS via ACE2 receptors. These receptors are found on the brain and spinal glia and in the vascular endothelium. Moreover, SARS‐CoV‐2 post‐infectious reaction could be a probable mechanism behind acute transverse myelitis. It is, therefore, necessary for clinicians to consider COVID‐19 besides other well‐known etiologies if they encounter patients with transverse myelitis even without preceding fever and cough. Further research is required to evaluate patients' responses to corticosteroid treatment and plasma exchange.

## CONFLICTS OF INTERESTS

The authors declare that there is no conflict of interest regarding the publication of this article.

## AUTHOR CONTRIBUTION

Soroor Advani: Drafting the manuscript and patient management. Seyyed Mohammad‐Mahdi Hosseini: Drafting the manuscript and English language revision. Alireza Zali, Reza Jalili Khoshnoud, Davood Ommi, and Alireza Fatemi: Scientific revision of manuscript. Farzad Ashrafi: Patient management, scientific revision of manuscript, and corresponding author.

## ETHICAL APPROVAL

Both patients were provided with information about the report and written informed consent was obtained.

## CONSENT

Consent for publication was obtained from both patients and is available from the corresponding author.

## Data Availability

The datasets used during the current study are available from the corresponding author on reasonable request.
